# SapTrap assembly of repair templates for Cas9-triggered homologous recombination with a self-excising cassette

**DOI:** 10.17912/W2KT0N

**Published:** 2018-05-01

**Authors:** Daniel J. Dickinson, Mark M. Slabodnick, Alicia H. Chen, Bob Goldstein

**Affiliations:** 1 Department of Biology and Lineberger Comprehensive Cancer Center, University of North Carolina at Chapel Hill, Chapel Hill, NC, USA; 2 Present address: Department of Molecular Biosciences, University of Texas at Austin, Austin, TX USA

**Figure 1.**
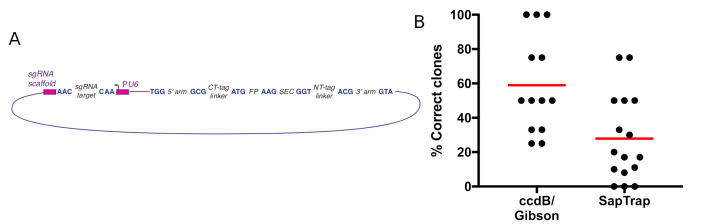
A) General organization of SapTrap-assembled constructs. Italics indicate the fragments that are assembled to build the final construct, and blue upper-case letters are the 3-nucleotide overlaps that enable ordered assembly. B) Efficiency of construct assembly (% correct clones) using SapTrap (Schwartz and Jorgensen 2016) or a ccdB/Gibson-based approach (Dickinson et al. 2015).

## Description

The advent of CRISPR/Cas9 technology in *C. elegans* has enabled an unprecedented level of control over this organism’s genome, which is facilitating research in a wide variety of fields. Several different experimental approaches exist for CRISPR in this organism (Dickinson and Goldstein 2016). One particularly powerful approach uses a drug selection with a Self-Excising Cassette (SEC) to isolate the desired genetically-modified animals following Cas9-triggered homologous recombination (Dickinson et al. 2015). The most labor-intensive step in this procedure is the construction of a plasmid-based repair template for homologous recombination.

SapTrap (Schwartz and Jorgensen 2016) is a high-throughput cloning procedure that allows modular assembly of repair templates for CRISPR/Cas9-triggered homologous recombination. The original publication that described SapTrap cloning (Schwartz and Jorgensen 2016) used *unc-119(+)* as a positive selection marker for isolating recombinant strains. Compared to *unc-119* selection, SEC selection is faster; can be used directly in a wild-type background; and eliminates an extra injection step followed by outcrossing to remove the selection markers after strain isolation (Dickinson et al. 2015). Because of these advantages of SEC selection over *unc-119*, we wished to incorporate the SEC into the SapTrap workflow. Here, we describe a toolkit of plasmids that allow building SEC-containing repair templates via SapTrap cloning.

In brief, SapTrap links pieces of a repair template construct together by means of unique 3-bp overlaps generated by the type II restriction enzyme SapI. Constructs generated using SapTrap have the general form shown in Figure 1A. In a typical assembly reaction, the sgRNA target is provided as a pair of oligos, the homology arms are provided as PCR products, and a Fluorescent Protein (FP), SEC and linker fragments are provided by pre-existing donor vectors. These fragments are mixed together, along with the requisite enzymes, and are assembled in a single reaction. The linkers can be omitted from the final construct if desired, by designing the homology arms with 3-bp overlaps that anneal directly to the FP or SEC instead of to the linker.

Using SapTrap to generate repair templates for SEC selection involves two modifications to the original approach described by Schwartz and Jorgensen. First, an AAG junction is added between the FP and SEC fragments (Figure 1A). Together, these two fragments replace the FP/unc-119(+) donors used by the Jorgensen lab. Second, it is important to ensure that a coding exon follows SEC in order to avoid nonsense-mediated decay. For this reason, we always include the NT-tag linker in our constructs, even for C-terminal tags.

We generated and tested fluorescent protein donors covering the current best available fluorescent proteins for *C. elegans* imaging (Heppert et al. 2016 and our unpublished observations) along with SEC donors containing three different *Lox* sites that do not recombine with each other, for multiplex editing. These constructs have been deposited at Addgene and are listed in Table 1.

Using various combinations of fluorescent protein, SEC and linker donors, we were routinely able to obtain the desired repair templates, although the cloning efficiency was lower compared to the ccdB/Gibson-based cloning approach described by Dickinson et al. (2015) (Figure 1B). Note that, for both the Gibson and SapTrap data reported in Figure 1B, we used PCR products 500-700 bp in length as the homology arms, enabling a direct comparison of the two cloning methods. We have not tested short homology arms formed from annealed oligonucleotides, which were used by Schwartz and Jorgensen (2016), with either cloning approach.

Based on our extensive experience with both SapTrap and the ccdB/Gibson-based cloning procedure, we believe that each procedure has advantages. The advantages of SapTrap are:

- SapTrap is more modular: different fluorophores, epitope tags, and selection cassettes can be freely combined without the need to construct a new FP–SEC vector each time.
- SapTrap does not require the use of ccdB-containing vectors, which are toxic to *E. coli* and therefore can be difficult to grow and maintain.
- The SapTrap procedure can be used to generate a single plasmid that contains both the repair template and sgRNA – that is, all of the unique components for a genome editing procedure. This saves time by eliminating the need to clone separate Cas9–sgRNA and repair template plasmids. Note, however, that including the sgRNA in the repair template is not a requirement; SapTrap can also be used to assemble repair templates that do not include the sgRNA. The repair template-only option is useful when one has already generated a separate Cas9–sgRNA construct, or when one wishes to control the concentration of the repair template and sgRNA separately. We always inject 50 ng/µL Cas9 and sgRNA, but often prefer to have the repair template at lower concentration because this results in fewer extrachromosomal arrays being formed.

Conversely, the ccdB/Gibson-based approach has the following advantages:

- In our hands, the ccdB/Gibson-based approach generates a higher fraction of correct clones (Fig. 1B). Although the difference may, at first glance, appear small, in practice it is significant. Using the ccdB/Gibson-based approach, we almost always obtain a correct clone by picking and sequencing four colonies; that is, at least 25% of clones are correct in nearly all cases. In contrast, just over half (9/16) of our SapTrap reactions have yielded less than 25% correct clones, so the direct sequencing strategy is impractical. Thus, SapTrap requires an extra step (screening clones by colony PCR and/or restriction digestion) that is unnecessary with the ccdB/Gibson-based approach.
- The ccdB/Gibson-based approach can be used even if the homology arms contain SapI sites.

Thus, these two cloning approaches are both valuable. We hope that, by making it possible to generate SEC-containing repair templates using either approach, our constructs will facilitate users choosing the best cloning protocol for a particular genome engineering application.

**Protocol**

A detailed protocol for construct design and SapTrap assembly is available on our website (http://wormcas9hr.weebly.com/protocols.html ) and will be kept continually updated.

## Reagents

Table 1 New vectors that have been deposited at Addgene:

**Table:** 

Plasmid Name	Insert	Description	Addgene Number
**​FP Donors**	Value	Value	Value
​pDD377	​mTurquoise2	Worm codon-optimized mTurquoise2	​91823
pDD372	​GFP	Worm codon-optimized GFP	​91824
​pMS050	​mScarlet-I	Worm codon-optimized mScarlet-I	91826
​pDD375	​mKate2	Worm codon-otimized mKate2	91825
pDD378	HaloTag	​Worm codon-optimized HaloTag	91827
pDD401	Pmyo-2::GFP	Pharyngeal promoter driving GFP. Intended for knocking out a gene and putting Pmyo-2::GFP in its place.	91828
​pDD397	​AID FP slot	​Auxin-inducible degron; intended for tagging a gene with AID but no fluorescent protein	91822
**SEC Donors**			
​pDD363	LoxP-SEC-LoxP	​SEC flanked by LoxP sites	91829
​pDD382	​Lox2272-SEC-Lox2272	SEC flanked by Lox2272 sites	91830
pDD364	Lox511I-SEC-Lox511I	SEC flanked by Lox511I sites	91831
**Other plasmids**			
pDD398	AID NT-tag linker	Auxin-inducible degron for the NT-tag linker slot	91832
pDD399	AID CT-tag linker	​Auxin-inducible degron for the CT-tag linker slot	91821​
pDD379		SapTrap destination vector that includes the more efficient F+E sgRNA scaffold (Ward 2015)	91834
pDD121	Cas9	Expression plasmid for Cas9 only (no sgRNA)	91833​
